# Characterization of the porcine synovial fluid proteome and a comparison to the plasma proteome

**DOI:** 10.1016/j.dib.2015.08.028

**Published:** 2015-09-04

**Authors:** Tue Bjerg Bennike, Omar Barnaby, Hanno Steen, Allan Stensballe

**Affiliations:** a Department of Health Science and Technology, Aalborg University, Fredrik Bajersvej 3B, 9220 Aalborg, Denmark; b Departments of Pathology, Boston Children's Hospital and Harvard Medical School, Boston, MA, USA

**Keywords:** Synovial fluid, Plasma, Proteomics, Post-translational modification, Human, Porcine, Pig, Optimization, Digestion

## Abstract

Synovial fluid is present in all joint cavities, and protects the articular cartilage surfaces in large by lubricating the joint, thus reducing friction. Several studies have described changes in the protein composition of synovial fluid in patients with joint disease. However, the protein concentration, content, and synovial fluid volume change dramatically during active joint diseases and inflammation, and the proteome composition of healthy synovial fluid is incompletely characterized.

We performed a normative proteomics analysis of porcine synovial fluid, and report data from optimizing proteomic methods to investigate the proteome of healthy porcine synovial fluid (Bennike et al., 2014 [1]). We included an evaluation of different proteolytic sample preparation techniques, and an analysis of posttranslational modifications with a focus on glycosylation. We used pig (*Sus Scrofa*) as a model organism, as the porcine immune system is highly similar to human and the pig genome is sequenced. Furthermore, porcine model systems are commonly used large animal models to study several human diseases.

In addition, we analyzed the proteome of human plasma, and compared the proteomes to the obtained porcine synovial fluid proteome. The proteome of the two body fluids were found highly similar, underlining the detected plasma derived nature of many synovial fluid components. The healthy porcine synovial fluid proteomics data, human rheumatoid arthritis synovial fluid proteomics data used in the method optimization, human plasma proteomics data, and search results, have been deposited to the ProteomeXchange Consortium via the PRIDE partner repository with the dataset identifier PXD000935.

## Graphical abstract

**Figure:**
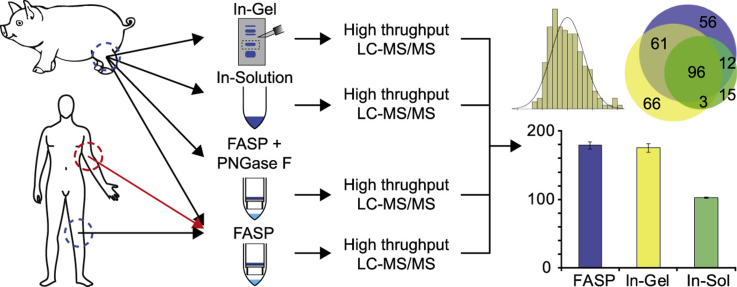

**Specifications table**

**Table:** 

Subject area	*Biology*
More specific subject area	*An analysis of the protein component of porcine synovial fluid, and a comparison to human plasma.*
Type of data	*Raw files and text/excel files*
How data was acquired	*Mass spectrometry liquid chromatography*
*Two different high-resolution/high-accuracy mass spectrometer*
*systems were used: TripleTOF 5600 (SCIEX) and Q Exactive*
*(Thermo Scientific)*
Data format	*Raw and analyzed data.*
Experimental factors	*Human and porcine synovial fluid as well as human plasma was analyzed.*
Experimental features	*Synovial fluid was digested using trypsin with in solution digestion, filter aided sample preparation, and in-gel digestion protocols. Plasma was digested using filter aided sample preparation. The purified peptides were analyzed by electrospray ionization liquid chromatography mass spectrometry.*
Data source location	*Steen & Steen Laboratory, Enders Research Building, Boston Children’s Hospital, 320 Longwood Ave, Boston, MA, USA.*
Data accessibility	The MS proteomics data have been deposited to the ProteomeXchange Consortium via the PRIDE partner repository with the dataset identifier PXD000935 [2–5]. Direct download link: http://www.ebi.ac.uk/pride/archive/projects/PXD000935

**Value of the data**

• Increase our knowledge of synovial fluid in healthy state, usable for future studies on joints and joint disease.
• Investigate the plasma-derived nature of many synovial fluid proteins.
• Identify differences and similarities between the porcine proteome and the human proteome, usable in assessing pigs as a model system for human diseases.
• Information regarding the proteome of synovial fluid from a healthy joint will form the basis for research in joint diseases, such as osteoarthritis and rheumatoid arthritis.

## 1 Experimental design

Synovial fluid is an ultrafiltrate of plasma, and the two body fluids share many similarities in terms of protein composition [6,7]. To investigate the protein component of healthy synovial fluid, we analyzed the proteome of synovial fluid from healthy porcine knee joints. We used pig as a model organism, as the pig proteome is similar to humans [1]. The protein concentration in synovial fluid from healthy knee joints is approximately 25 mg/mL, and albumin constitutes approximately 12 mg/mL [8–12]. Because high-abundant proteins might hinder the identification of lesser abundant ones, most work conducted on synovial fluid has employed immunodepletion strategies and/or gel-based separation techniques [1]. However, in-gel digestion strategies are, while robust, typically not compatible with high-throughput proteome analyses [13–15]. Therefore, we investigated the use of alternative high-throughput protein identification and quantitation proteomics methods, utilizing digestion method optimization and two *state-of-the-art* mass spectrometers, to increase the number of identified synovial fluid proteins.

## 2 Materials and methods

### 2.1 Collection of synovial fluid samples

We utilized six adolescent Yucatan minipigs (Coyote CCI, Douglas, MA, USA), aged 12–15 months for the study. The minipigs were housed and monitored by the Animal Resources at Boston Children's Hospital and handled according to the Institutional Animal Care and Use Committee protocols.

Following acclimation to the environment for at least three days, synovial fluid was extracted using a needle. When necessary to facilitate the extraction, 3 mL sterile saline was injected into the joint, which was bend ten times. Additionally, synovial fluid was obtained from a rheumatoid arthritis patient according to an approved IRB protocol (IRB-P00006443). To remove cells and cell debris, the samples were centrifuged at 3.000*g* at room temperature for 10 min, and the supernatants were stored at −80 °C. The protein concentration was estimated for normalization of sample material using a bicinchoninic acid assay kit (Bio-Rad, Hercules, CA, USA) according to manufacturer's instructions.

### 2.2 Synovial fluid protein digestion

To increase the number of identified proteins we evaluated and combined data from three trypsin digest protocols.

1. Filter-Aided Sample Preparation (FASP) Digestion: 90 µg total synovial fluid protein was digested using a FASP digestion kit (Protein Discovery, San Diego, CA, USA) according to manufacturer's instructions with 30 kDa molecular weight cutoff spin filters. To assess the need of glycan removal when working with synovial fluid peptide-N4-(N-acetyl-beta-glucosaminyl)-asparagine amidase (PNGase F) (New England BioLabs, Ipswich, MA, USA) was added to these samples prior to the trypsin digestion step according to manufacturer's instructions, and the samples were incubated overnight at 37 °C, after which trypsin was added and the FASP protocol was resumed.
2. In-gel digestion: three samples of 150 µg total synovial fluid protein was prepared for SDS-PAGE with Laemmli Sample Buffer (Bio-Rad, Hercules, USA) according to manufacturer’s instructions. The sample was fractionated using *NuPAGE* 4–12% Bis-Tris mini gels (Invitrogen) at 150 V for 65 min in MOPS SDS-running buffer (Invitrogen). The gel was stained using Coomassie blue SimplyBlue SafeStain (Invitrogen) according to manufacturer’s instructions. Three gel-lanes were loaded with 150 µg total synovial fluid protein, each divided into 10 sections, and subjected to standard in-gel tryptic digestion as previously described [16–18].
3. In-solution digestion: performed according to Gallien et al. [19]. 90 µg total synovial fluid protein was diluted with 8 M urea 100 mM ammonium bicarbonate to a final volume of 25 µL. The sample was reduced with dithiothreitol at a final concentration of 12 mM for 30 min at 37 °C, and alkylated with iodoacetamide at a final concentration of 40 mM for 1 h at room temperature in the dark. The samples were diluted with 0.1 M ammonium bicarbonate to a total volume of 100 µL, 2 µg trypsin was added and the sample was digested overnight at 37 °C.

For all digestion methods, a 50:1 (w/w) ratio protein to sequencing grade modified trypsin (Promega, Fitchburg, MA, USA) was added, and the samples were digested overnight at 37 °C. After trypsin digestion, the samples were desalted with TARGA C18 columns (Nest Group, Southborough, MA, USA), dried in a vacuum centrifuge, and the dry product was stored at −80 °C. Prior to liquid chromatography tandem mass spectrometry (LC-MS/MS) analysis, the peptides were resuspended in 5% acetonitrile (ACN), 5% formic acid (FA).

### 2.3 Human plasma sample preparation

Human plasma was acquired for an ongoing method optimization study using an anonymized, discarded plasma sample, and is therefore not considered research of human subjects. Hundred µg plasma protein was digested using the FASP protocol, modified to use 10 kDa molecular weight cutoff spin filters. The proteins were digested overnight at 37 °C, using a 25:1 (w/w) ratio of proteins to trypsin/LysC mix (Promega, Madison, WI, USA). The peptides were desalted with Oasis HLB columns (Waters, Milford, MA, USA), fractionated into 12-fractions by isoelectric focusing on a 3100 OFFGEL fractionator (Agilent, Santa Clara, CA, USA). The resulting fractions were desalted using Oasis HLB columns (Waters, Milford, MA, USA) and stored at −80 °C. Prior to LC-MS/MS analysis, the peptides were resuspended in 2% ACN, 0.1% FA.

### 2.4 LC-MS/MS analysis

To increase the number of identified synovial fluid proteins, two different high resolution/high accuracy mass spectrometer systems were used for the discovery-based proteomic experiment:

1. A TripleTOF 5600 (SCIEX, Framingham, MA, USA) connected online with a nanoflow UPLC and a NanoFlex system (Eksigent/SCIEX). The samples were loaded onto a 15 cm reversed phase C18 200 µm chip with 2 µL/min in 100% solvent A (0.1% FA). The samples were then separated using a 15 cm reversed phase C18 75 µm chip, and eluted with a linear gradient of 2% solvent B (0.1% FA in ACN) which was raised to 35% solvent B over 120 min (60 min for in-gel digested samples) at a constant flow rate of 500 nL/min.
2. A Q Exactive (Thermo Scientific, Waltham, USA) connected online to an EASY-nUHPLC 1000 (Thermo Scientific). The samples were loaded onto a 10.5 cm reversed phase C18 PicoChip column (Picochip, Bath, England) with a flow rate of approximately 1 µL/min in 98% solvent A and 2% solvent B, and eluted with eluent B using a linear gradient which was raised to 35% over 120 min at a constant flow rate of 300 nL/min.

### 2.5 Proteomic data analysis

The SCIEX.wiff data-files were analyzed using ProteinPilot 4.5 (Rev. 1656, Paragon Algorithm 4.5.0.0). To identify the most commonly single observed PTMs, data-files were searched in thorough-mode with a focus on biological modifications in ProteinPilot to include 303 different PTMs, and the result was assessed using ProteinPilot Descriptive Statistics Template v3.001.

The.raw data-files from synovial fluid analyzed on the Q Exactive were searched using MaxQuant 1.4.1.2. [20]. All standard settings were employed with carbamidomethyl(C) as a static modification and deamidation (NQR), oxidation (M), and protein N-terminal acetylation were included as variable modifications. Label-free quantitation of all proteins was performed in MaxQuant based on integrated precursor intensities. The human plasma samples was searched using Mascot v2.3.02 with identical modification settings to MaxQuant, parent tolerance of 10 ppm, and fragment tolerance of 0.03 Da.

Porcine synovial fluid data was searched against the Uniprot *Sus scrofa* reference proteome database (downloaded 11/09/2013, containing 26,070 entries). The rheumatoid arthritis patient synovial fluid data was searched against all reviewed *Homo sapiens* Uniprot proteins (downloaded 08/10/2013, containing 20,277 entries). The human plasma sample was searched against the Uniprot human database (R2001_05_crp_T, containing 35,806 entries). All proteins and peptides are reported below a 1% false discovery rate (FDR) cutoff.

## 3 Data

The proteomics data and result-files from the analysis have been deposited to the ProteomeXchange Consortium via the PRIDE partner repository with the dataset identifier PXD000935 [2–5], and can be downloaded directly (http://www.ebi.ac.uk/pride/archive/projects/PXD000935). Table 1 shows the list of submitted proteomics raw-datafiles and information regarding sample types, species, digestion protocol, and MS system. Table 2 shows the list of submitted analysis result files and a short description of the content. In our data, the FASP protocol was the most efficient, yielding the highest number of identifiable proteins in synovial fluid. Therefore, we conducted a comprehensive analysis of synovial fluid from six different pigs, analyzed in triplicates on a Q Exactive system. In addition, we conducted an analysis of human plasma, and the data demonstrates the high degree of similarity between plasma and synovial fluid, and the plasma-derived nature of many synovial fluid components. Many of these components were further identified in the full research paper [1].

## Funding sources

The Lundbeck Foundation Denmark (R124-A11485 and R181-2014-3372) and Carlsberg Foundation Denmark (CF14-0561) are acknowledged for grants enabling the analysis (TBB grants). The overseas collaboration was supported by the Knud Hojgaard Foundation Denmark (j.nr. 45.462), the Oticon Foundation Denmark and the Otto Monsteds foundation Denmark (12-70-0953) (TBB grants). This investigation was supported by National Institutes of Health under NIAMS AR054099 (MMM) and AR050180 (MLW), and the Ruth L. Kirschstein National Research Service Award (F32 AR061186) (CMH). The content is solely the responsibility of the authors and does not necessarily represent the official views of the National Institutes of Health.

## Figures and Tables

**Table 1:** Description of file-names in the ProteomeXchange repository PXD000935. 5600 (.wiff) MS files were searched using ProteinPilot, and Q Exactive (.raw) MS files using MaxQuant. The number of identified proteins is reported.

**File**	**Sample type**	**Species**	**Digestion**	**MS system**	**# ID proteins**
121029_TB_FD1	SF	Porcine	FASP	5600	191
121029_TB_FD7	SF	Porcine	FASP	5600	182
121029_TB_FD9	SF	Porcine	FASP	5600	164
121029_TB_UD1	SF	Porcine	In-solution	5600	103
121029_TB_UD7	SF	Porcine	In-solution	5600	104
121029_TB_UD9	SF	Porcine	In-solution	5600	101
121115_TB_D1_ingel# (fraction 1–10)	SF	Porcine	In-gel	5600	192
121115_TB_D7_ingel# (fraction 1–10)	SF	Porcine	In-gel	5600	173
121115_TB_D9_ingel# (fraction 1–10)	SF	Porcine	In-gel	5600	161
121113_TB_HumanSF1_6	SF	Human	FASP	5600	183
121113_TB_HumanSF1_7	SF	Human	FASP	5600	183
121113_TB_HumanSF2_8	SF	Human	FASP	5600	172
121113_TB_HumanSF2_9	SF	Human	FASP	5600	154
130103_TB_SFGlycotest_SFDeglyco_1	SF	Porcine	PNGase F, FASP	5600	189
130103_TB_SFGlycotest_SFDeglyco_2	SF	Porcine	PNGase F, FASP	5600	184
130103_TB_SFGlycotest_SFUntreated_1	SF	Porcine	FASP	5600	184
130103_TB_SFGlycotest_SFUntreated_2	SF	Porcine	FASP	5600	189
NeatPlasma_2hr	Plasma	Human	FASP	Q Exactive	200
Tue_SF_FASP_143 (three replicates)	SF	Porcine	FASP	Q Exactive	242
Tue_SF_FASP_155 (three replicates)	SF	Porcine	FASP	Q Exactive	204
Tue_SF_FASP_165 (three replicates)	SF	Porcine	FASP	Q Exactive	231
Tue_SF_FASP_168 (three replicates)	SF	Porcine	FASP	Q Exactive	250
Tue_SF_FASP_37 (three replicates)	SF	Porcine	FASP	Q Exactive	164
Tue_SF_FASP_52 (three replicates)	SF	Porcine	FASP	Q Exactive	227

**Table 2:** Description of search file-names in the ProteomeXchange repository PXD000935.

**Search files filename**	**Description of content**
[Filename]_PeptideSummary.txt	ProteinPilot exported list of peptides.
[Filename] _ProteinSummary.txt	ProteinPilot exported list of proteins.
[Filename] __FDR.xlsx	ProteinPilot FDR analysis.
Transcriptome MQ Combined PorcineSF.zip	MaxQuant result folder “combined” of Q Exactive porcine synovial fluid samples, search against a transcriptome database of the synovial membrane, and thus contains a list of synovial fluid proteins expressed by the synovium [1].
UniProt MQ Combined Plasma Human.zip	MaxQuant result folder “combined” of human plasma, searched against the human Uniprot database, and thus contains a list of all identified human plasma proteins.
UniProt MQ Combined SF PorcineSF.zip	MaxQuant result folder “combined” of Q Exactive porcine synovial fluid samples, search against the porcine Uniprot database, and thus contains a list of all identified synovial fluid proteins.
